# Metabolism and Ovarian Function in PCOS Women: A Therapeutic Approach with Inositols

**DOI:** 10.1155/2016/6306410

**Published:** 2016-08-04

**Authors:** Antonio Simone Laganà, Paola Rossetti, Massimo Buscema, Sandro La Vignera, Rosita Angela Condorelli, Giuseppe Gullo, Roberta Granese, Onofrio Triolo

**Affiliations:** ^1^Unit of Gynecology and Obstetrics, Department of Human Pathology in Adulthood and Childhood “G. Barresi”, University of Messina, 98125 Messina, Italy; ^2^Unit of Diabetology and Endocrino-Metabolic Diseases, Cannizzaro Hospital, 95126 Catania, Italy; ^3^Department of Clinical and Experimental Medicine, Research Centre of Motor Activity and Metabolic Rehabilitation in Diabetes (CRAMD), University of Catania, 95124 Catania, Italy

## Abstract

Polycystic ovary syndrome (PCOS) is characterized by chronical anovulation and hyperandrogenism which may be present in a different degree of severity. Insulin-resistance and hyperinsulinemia are the main physiopathological basis of this syndrome and the failure of inositol-mediated signaling may concur to them. Myo (MI) and D-chiro-inositol (DCI), the most studied inositol isoforms, are classified as insulin sensitizers. In form of glycans, DCI-phosphoglycan and MI-phosphoglycan control key enzymes were involved in glucose and lipid metabolism. In form of phosphoinositides, they play an important role as second messengers in several cellular biological functions. Considering the key role played by insulin-resistance and androgen excess in PCOS patients, the insulin-sensitizing effects of both MI and DCI were tested in order to ameliorate symptoms and signs of this syndrome, including the possibility to restore patients' fertility. Accumulating evidence suggests that both isoforms of inositol are effective in improving ovarian function and metabolism in patients with PCOS, although MI showed the most marked effect on the metabolic profile, whereas DCI reduced hyperandrogenism better. The purpose of this review is to provide an update on inositol signaling and correlate data on biological functions of these multifaceted molecules, in view of a rational use for the therapy in women with PCOS.

## 1. PCOS Definition(s): The More, the Better?

The polycystic ovary syndrome (PCOS) is a clinical syndrome characterized by chronic anovulation and hyperandrogenism [1], affecting 6–10% [2] of women in reproductive age. Its etiology is complex, heterogeneous, and not completely understood. Current evidence suggests that genetic, endocrine, metabolic, and environmental aspects are involved in determining the syndrome.

There are three recognized definitions to diagnose PCOS, among which the Rotterdam Criteria proposed in 2003 by European Society of Human Reproduction and Embryology/American Society for the Reproductive Medicine (ESHRE/ASRM) are the most recommended [1, 2]. The Rotterdam Criteria consider at least two of three criteria between clinical or biochemical hyperandrogenism, menstrual irregularity, and polycystic ovaries characterized by ultrasound detection of 12 or more follicles <9 mm in diameter and/or increased ovarian volume >10 mL and in absence of a dominant follicle [3]. In 1990 the National Institutes of Health (NIH) proposed that PCOS might be diagnosed with hyperandrogenism and irregular menstrual cycles without knowledge of ovarian ultrasound pattern [4]. Instead, the Androgen Excess PCOS Society [5] recommends diagnosis in the presence of hyperandrogenism plus one of two other criteria among ovulation dysfunction and PCO morphology, according to Rotterdam Criteria (Table 1). Nevertheless, all three definitions underline the importance to exclude other clinical disorders that may mimic PCOS.

Many aspects of this syndrome are still debated; for example, which criteria may be adopted to make a diagnosis during adolescence? The third PCOS consensus workshop in 2010 identified gaps in knowledge and dealt with these controversial aspects [6]. During adolescence as many as 85% of menstrual cycles are anovulatory during the first years following menarche and as many as 59% are anovulatory up to three years after the menarche in physiological conditions [7]. Furthermore only 40% of adolescents with irregular cycles display polycystic ovary on ultrasound [8]. On the other hand, other authors found in adolescent population a high prevalence of characteristic polycystic ovaries in otherwise asymptomatic girls, suggesting a high prevalence of this feature in this age range [9]. In addition, hyperandrogenism seems a most important feature to lead diagnosis during adolescence [10]. Basing on these data, it was suggested that all three criteria should be present to diagnose PCOS in adolescents and that irregularity in menstrual cycles must persist for at least 2 years after menarche [11].

## 2. PCOS and Metabolic Aspects: The Key Role of Insulin-Resistance

Insulin-resistance and hyperinsulinemia are tightly related to pathogenesis of PCOS [12, 13] which may be exacerbated by coexistence of obesity, affecting about 50% of women with PCOS [14]. Nevertheless, many studies showed that insulin-resistance affected also normal weight women with PCOS [15, 16]. Besides, in lean patients with PCOS the visceral adiposity seems to be greater than in nonobese women without PCOS and this feature might in part explain metabolic disorders in these patients [16].

Overall between 50 and 70% of women with PCOS had demonstrable insulin-resistance [5], which results in a compensatory increase in insulin secretion by *β*-islet cells of the pancreas. However, many women with insulin-resistance and PCOS also had impaired *β* cell function and the grade of dysfunction seems to be related to family history of type II diabetes [17]. Furthermore, hyperinsulinemic insulin-resistance stimulates the ovarian androgen production and increases the likelihood to develop type 2 diabetes, metabolic syndrome, and cardiovascular disease [18].

About 22% of women under 20 years and 53% between 30 and 39 years of age affected by PCOS met the criteria for metabolic syndrome (MS) according to the National Cholesterol Education Program Adult Treatment Panel III (NCEP-ATP III) [19]. Indeed, the prevalence of MS in patients with PCOS seems to be 2-fold higher than reported for age-matched general population even when stratified by both age and Body Mass Index (BMI). Basing on these epidemiological data, it is possible that the presence of PCOS by itself conferred an increased risk of MS, since the intrinsic insulin-resistance of PCOS patients [20].

Other authors [21] found a higher risk of MS in young patients with PCOS compared with general age-matched population such as 37% versus 5% and 47 versus 13%, according to two different criteria proposed for diagnosis of MS [22, 23]; furthermore among obese young patients the prevalence increased to 63%. Obesity and insulin-resistance were significant risk factors to develop MS, but hyperandrogenemia remained a significant predictor of MS after adjusting for both obesity and insulin-resistance: in fact the prevalence of MS was higher in patients with PCOS and higher bioavailable testosterone levels [21]. The prevalence of type 2 diabetes in women with PCOS was 8%, 8-fold greater than general population, and prevalence of impaired glucose tolerance (IGT) and impaired fasting glucose (IFG) was 11% and 0.9%, respectively. According to the 2003 guidelines of the Expert Committee on the diagnosis and classification of diabetes mellitus [24], which established for IFG diagnosis fasting glucose blood levels higher than 100 mg/dL, the prevalence of IFG was 10% [20]. In others studies the prevalence of type 2 diabetes was similar, from 4 to 10% of women with PCOS [25].

Dyslipidemia is one of most frequent features in patients with PCOS. Hyperinsulinemia and insulin-resistance are related to abnormality of lipid profile, particularly to decreased levels of high density lipoprotein cholesterol (HDL-C) levels and increased levels of triglycerides and of small dense low density lipoprotein cholesterol (LDL-C) [26]. Insulin-resistance causes a rise in free fatty acid (FFA) plasma levels due to increased synthesis from liver and increased mobilization from adipose tissue. The excess of FFA leads* per se* to insulin-resistance by inactivation of key enzymes such as pyruvate dehydrogenase (PDH) or by decreasing glucose transport activity, which may be a consequence of altered insulin signaling through decreased insulin receptor substrate-1 (IRS-1) associated PI3 kinase activity [27]. The Hoorn study, started in 1989 and finished in 1998 after a 10 years' follow-up, has already registered increased cardiovascular diseases (CVD) mortality among patients with MS, by 1.8- and 1.2-fold, respectively, in men and women, and all-causes mortality was increased by 1.5-fold in both sexes. Furthermore, patients with MS were 2.9-fold more likely to develop diabetes [28].

A pioneering study by Burghen et al. [12] identified a relationship between hyperandrogenism and hyperinsulinemia in polycystic ovary disease. Since those first studies it became evident that insulin-mediated glucose disposal, calculated with euglycemic glucose clamp, was decreased in PCOS as a result of insulin-resistance in skeletal muscle and fat tissue. Hepatic glucose output and its insulin-mediated suppression were also altered, consistent with hepatic insulin-resistance [29, 30]. Other authors showed that adolescents with PCOS and with IGT had the same insulin sensitivity compared to adolescents with normal glucose tolerance but showed higher hepatic glucose output and 50% lowering in the first phase of insulin secretion [31]. Other studies confirmed a decrease in both first and second phases of insulin response to glucose, suggesting abnormal *β*-cell function, in women with PCOS and abnormal glucose tolerance [17].

## 3. PCOS and Impaired Follicular Maturation

PCOS often develops during puberty inducing dermatological signs (hirsutism, acne, and alopecia), irregular menses, and biochemical alterations associated with high levels of testosterone, DHEA, androstenedione, and luteinizing hormone (LH) and increased LH/follicle-stimulating hormone (FSH) ratio, together with a concurrent reduction of sex hormone binding globulin (SHBG) and insulin-like growth factor (IGF) binding protein [32]. These alterations are due to insulin-resistance and hyperinsulinemia. Hyperinsulinemia stimulates pituitary LH release (thus increasing LH/FSH ratio), raises androgen production from ovarian theca cells, and decreases SHBG synthesis, leading to enhanced free testosterone levels. Recent studies showed that hyperandrogenism may also be due to local inflammatory response of ovarian theca cells to reactive oxygen species [33] or to cytokines and chemokines produced by dysfunctional adipose tissue [34]. In addition, obesity interferes with the hypothalamus-pituitary-ovary regulation system and therefore inhibits the physiological process of ovarian follicular maturation [35]. Overall, hyperandrogenism together with high levels of LH significantly disturbs the physiological process of ovarian follicular maturation and may lead to anovulatory cycles.

## 4. Rationale of Inositol-Based Therapeutic Approach in PCOS

Current evidence suggests that insulin-resistance and secondary hyperinsulinemia play an important role in hyperandrogenism, anovulation or irregular cycles, and metabolic alteration in both lean and obese patients with PCOS. The current therapy aims to improve insulin-resistance, to reach a reduction of compensatory hyperinsulinemia and then improve metabolic and ovulatory features in patients with PCOS. According to recent guidelines, insulin-sensitizer drugs are the first line therapy in women with metabolic abnormalities and irregular cycle with the purpose to improve fertility, whereas a lifestyle change with weight loss and physical activity is the first step in overweight and obese PCOS patients. Metformin is recommended both in patients with metabolic abnormalities (such as IGT, IFG, and type 2 diabetes) and in women who desire pregnancy, as an adjuvant therapy for infertility and to decrease the risk of ovarian hyperstimulation syndrome [36].

In the past, many researchers centered their studies on the role played by inositols as second messengers in insulin signaling transduction [37]. In the last two decades, inositols were found to have important effects on ovulation and metabolism [38]; other studies underlined the importance of myo-inositol (MI) in oocyte differentiation and inositol ability to improve fecundation with* in vitro* fertilization techniques in women with PCOS [39].

Today we know much more about the key role that MI and D-chiro-inositol (DCI) play in insulin signaling pathway and the crucial function of myo-inositol in oocyte maturation. Inositols belong to a family of nine stereoisomeric six-carbon cyclitols which includes myo, muco, neo, scyllo, and D- and L-chiro-inositol and other three cis, allo, and epi-inositol that do not exist in nature. MI is the most widely distributed in nature; the main source of MI is the diet, as it is found in a wide variety of foods such as whole wheat, seeds, and fruits. Inositol is assumed to be an essential B complex vitamin; however it is known that inositol can be synthesized in human body. The synthesis starts from glucose 6-phosphate (G6P), which is turned into inositol-3-phosphate and later dephosphorylated by inositol monophosphatase 1, yielding the free MI [40]. MI is then converted in DCI by epimerase in mammalian tissues.

Inositol plays a fundamental role in the cell in two different manners: (1) incorporated in membrane phospholipids, producing phosphoinositides upon membrane receptor stimulation [41] and (2) in form of inositolphosphoglycans (IPG) that can be located at the inner or outer side of the plasma membrane and are involved in insulin transduction signaling as second messengers [42].

Inositol phospholipids have long been known as fundamental components of cellular membranes, concentrated at the cytosolic surface, and have a crucial function in membrane integrity as well as in intracellular signaling. Phosphatidylinositol (PtdIns) is a precursor of arachidonic acid, an intermediate in prostaglandins synthesis. PtdIns is also a precursor of phosphoinositides by reversible phosphorylation of the inositol ring in different positions (3,4,5), resulting in generation of seven phosphoinositide species [41], among which phosphatidylinositol 4P (PtdIns-4P) and phosphatidylinositol 4,5 bisphosphate (PtdIns 4,5P) are the most studied.

PtdIns-4P takes part in the regulation of cytoskeleton structure and PtdIns-4,5P is involved in regulation of linkage among actin and cytoskeleton with role in regulation of cellular motility; either have an important role in cellular and subcellular architecture [41].

PtdIns-4,5P is a key molecule in the “phosphoinositides signaling pathway” of G protein-coupled receptors (GPCRs) associated to phospholipase C, which generate the second messengers diacylglycerol (DAG) and inositol triphosphate (Ins 1,4,5-P). DAG in turn activates protein kinase C (PKC), an enzyme involved in many cell functions (proliferation, apoptosis, and differentiation), whereas Ins 1,4,5-P stimulates Ca^2+^ release from intracellular stores, activating several Ca^2+^-binding proteins and inducing numerous related physiological effects [43]. Some authors showed that Ins 1,4,5P played an important role in physiopathology of oocytes fertility, triggering the Ca^2+^ rise and secondary activation phenomena at fertilization [44].

In the last thirty years, MI and DCI attracted a growing interest since the discovery of their role in insulin-activated signaling pathways and in the physiopathology of metabolic syndrome and type II diabetes. Insulin stimulates glucose uptake from bloodstream and glucose oxidative and nonoxidative disposal by activation of glycogen synthase (GS) and mitochondrial PDH. The dominant paradigm involves the activity of the insulin receptor tyrosine (Tyr) kinase and its primary phosphorylated substrate, the insulin receptor substrate (IRS) family [45]. However, a number of studies have shown that transduction signaling activated by insulin receptors also involves cytoplasmic second messengers generated in parallel with the phosphorylation events initiated by Tyr kinase [46].

Two separate IPG were purified from rat liver: one (containing DCI and galactosamine) was able to activate pyruvate dehydrogenase phosphatase and the second (containing MI and glucosamine) was able to inhibit cyclic adenosine monophosphate (cAMP) kinase and adenylate cyclase, both regulatory enzymes in FFA metabolism [47].

Pioneering studies identified inositol glycans generated from* Trypanosoma brucei*, which displayed insulin mimetic action inhibiting lipolysis, glucose 6 phosphatase, and fructose-1,6 diphosphatase, well-known insulin-mediated metabolic effects [48].* In vivo* and* in vitro* studies later confirmed this hypothesis, showing that inositol glycans had insulin mimetic effects in human ovarian theca cells and stimulated testosterone biosynthesis [49, 50].

A recently proposed hypothesis is that insulin activates its specific Tyr kinase receptor which autophosphorylates itself and recruits IRS proteins, membrane adaptor proteins coupling insulin receptors to their intracellular signaling mechanisms. The first target of this pathway is phosphatidylinositol-3 kinase (PI3K), generating phosphatidylinositol 1,4,5 triphosphates (PIP3) which, in turn, activates the phosphoinositide-dependent kinase (PDK) kinase to phosphorylate and activate Akt kinase, leading to glucose transporter type 4 (GLUT4) translocation, GS activation, and stimulation of mammalian target of rapamycin (mTOR) kinase. At the same time, insulin receptor is coupled to the G-protein Gq that activates a phospholipase allowing the release of second messengers from GPI lipids (DCI glycan). These second messengers then activate phosphatase 2C*α* (PP2C*α*), which directly dephosphorylates and activates glycogen synthase and indirectly activates GS via PI3K. The same messengers inside mitochondria might activate pyruvate dehydrogenase phosphatase (PDHP), thereby activating pyruvate dehydrogenase and oxidative glucose metabolism [51].

Consistent with the proposed role of chiro-inositol as second messengers in insulin-mediated effects, other studies have shown that in muscle and urinary samples from type II diabetic patients DCI levels were about 50% lower than in samples from control subjects, whereas MI levels were not significantly different from control [52]. The difference observed in type II diabetes samples, with lower DCI and higher MI levels, was called inositol imbalance. The low DCI level and the inositol imbalance seemed to be related to the amount of insulin-resistance, worsening from normal to impaired glucose tolerance to type 2 diabetes [53]. Insulin-resistance was inversely related to urinary D-chiro-inositol excretion. Interestingly, women with PCOS had a blood deficiency of DCI and normal MI levels compared to control subjects [54]. The decreased production of chiro-inositol glycans observed during insulin-resistance might be explained by either deficiency of chiro-inositol or by decreased release mechanism [51]. On the other side, inositol imbalance might be partially explained with epimerase function failure: in fact, in a rat model of type II diabetes, conversion from MI to DCI in insulin-sensitive tissues was found to be reduced from 20 to 30% to 5% compared to normal rats [55]. Epimerase is also controlled by insulin that stimulates this enzyme to synthetize DCI starting from MI [56].

MI has been suggested to play a key role in oocyte fertilization: different studies describe a correlation between oocyte quality and follicular fluid concentrations of myo-inositol both in* in vitro* mouse models and on human oocytes studied during* in vitro* fertilization procedures [39, 57].

Intracellular calcium release mechanisms are accurately modulated during oocyte maturation and maximal sensitivity of calcium release is acquired during final stage of oocyte maturation. The PtdIns signaling pathway through inositol triphosphate (insP3) seems to regulate this oocyte maturation stage [58, 59]. Depletion of inositol may desensitize PtdIns by slowing down the resynthesis of precursors, as proposed by some authors [60].

The ovaries are sensitive to insulin and according to DCI ovary paradox theory, increased DCI level, due to increased epimerase function within the ovaries, is associated with a local MI deficiency and poor oocyte quality [61]. This failure had negative effects in FSH stimulation and in ovulation. Some authors showed that high (unnecessary?) dosage of DCI supplementation may damage oocytes [62]. In accordance with this finding, the conclusions of the “International Consensus Conference on Myo-inositol and D-Chiro-inositol in Obstetrics and Gynecology” [63] also emphasized the negative effect of increasing doses of DCI on the ovary. However, the upper limit for DCI may prove to be higher in future studies if MI is supplemented simultaneously with DCI.

## 5. Clinical Data

The use of inositol administration to patients with PCOS found a rationale in the numerous physiological functions that these molecules regulate, as discussed above.

In literature, a complete consensus was not reached about inositols dosage to be used and which inositol isoforms are more active to improve symptoms and biochemical rates in polycystic ovary syndrome. Another issue concerns the need for different therapeutic approaches according to principal signs and symptoms that the patient complains from, particularly if she wants to improve ovulation or ameliorate metabolic alteration or also reduce dermatological symptoms related to hyperandrogenism. One of the first studies carried out in overweight and obese patients with PCOS showed that the administration of DCI 1200 mg once daily for six to eight weeks led to a reduction of testosterone level, improved metabolic parameters, decreased insulin response to orally administered glucose, ameliorated systolic and diastolic blood pressure, triglycerides level, and ovulatory function [38]. In addition, our group recently confirmed these results, showing improved metabolic parameters, androgen levels, and dermatological sign of hyperandrogenism [64].

We already showed that the administration of either MI (4 g/die) or DCI (1 g/die) together with folic acid to two different groups of PCOS patients showed in both groups an improved systolic blood pressure, a reduction in circulating androgens levels, a reduction in LH and LH/FSH ratio, an increased insulin sensitivity evaluated through decreased HOMA index, and increased SHBG. According to our data analysis, we observed that MI seemed to have the most marked action on metabolic profile, whereas DCI mostly affected hyperandrogenism parameters [65]. Furthermore, both groups showed ameliorated regularity in menstrual cycles without any significant difference between the two inositol isoforms.

Other authors confirmed that administration of MI (2 g daily) plus folic acid in overweight women with PCOS improved insulin-sensitivity, biochemical hyperandrogenism and regularity of menstrual cycles [66] similar to the effect obtained using insulin sensitizers such as metformin [67], congruently with previous studies which showed recovery of menstrual cycles and a reduction in insulin plasma level also after oral glucose tolerance test [68]. MI was also shown to improve oxidative stress in erythrocytes, in addition to improving metabolic and biochemical parameters in insulin-resistant women with normal weight and PCOS [15].

Other studies compared administration of MI (4 g daily) alone versus MI + DCI (3300 mg plus 84 mg) showing an improvement of metabolic markers in both groups, although the improvement was better in MI + DCI group [69, 70].

A recent review from our research group was proposed to evaluate the optimal dosage and which combination of MI and DCI should be used as a supplement in patients with PCOS according to a capillary analysis of literature [71]. Both MI alone (administered at a dosage from 2 g to 4 g daily) and DCI alone improved metabolic parameters related to insulin-resistance in patients with PCOS, regularized menstrual cycles, and reduced androgen levels and related symptoms. For a complete and comprehensive summary of the evidence reported in this chapter refer to Table 2.

## 6. Conclusion

Polycystic ovary syndrome is frequently associated with insulin-resistance and hyperinsulinemia. Insulin-resistance is correlated to genetic factors, environmental factors, and hormonal status. From literature, it is known that PCOS benefits from insulin-sensitizer therapy such as metformin.

Umpteen studies in the last thirty years have studied inositols and their role in cellular biology. These molecules take part in insulin transduction signaling pathway through generation of inositol phosphoglycans (IPG), particularly DCI-IPG, which act as second messengers with insulin-like functions. Inositols play a key role in many cellular functions as phosphatidylinositide production in response to hormonal-receptor binding (i.e., insulin and FSH) two important second messengers, DAG and insP3, which are involved in numerous cellular processes regarding differentiation and oocyte maturation. Besides, phosphoinositides are involved in many other functions such as the control of cellular structure and motility. In patients with PCOS, DCI is reduced, concurring to insulin-resistance and worsening the metabolic features of these patients. On the other side, reduced levels of MI impair oocyte quality, interfering with physiological follicular maturation.

Consistent with these findings, restoring inositols levels with oral supplementations ameliorates insulin-resistance, hyperandrogenism, regularity of menstrual cycles, and oocyte quality in patients with PCOS. Nevertheless, we strongly solicit future studies based on large cohorts, in order to clarify the pivotal role of inositol's isoforms in addressing the hormonal and metabolic parameters toward homeostasis in PCOS patients. In addition, we take the opportunity to propose a “tailored” dosage, based on the pretreatment conditions, which may allow us to improve the current knowledge about long-term outcomes in this kind of patients.

## Figures and Tables

**Table 1:** Diagnostic criteria of PCOS according to different definitions.

	NIH (1990) necessary 2 criteria	Rotterdam criteria (2003) necessary at least 2 criteria	Androgen Excess PCOS Society (2009) necessary at least 2 criteria
*Clinical hyperandrogenism or biochemical hyperandrogenism*	Obligatory presence	Possible presence	Obligatory presence

*Oligo/anovulation *may manifest with frequent or infrequent bleeding, respectively, at <21 days or >35 days Rarely cycles may be anovulatory despite regular bleeding	Obligatory presence	Possible presence	Possible presence

*Ultrasound polycystic ovarian features:* presence of 12 or more follicles 2–9 mm in diameter and/or increased ovarian volume >10 mL (without dominant follicle) in either ovary	—	Possible presence	Possible presence

**Table 2:** Summary of the reported studies about myo-inositol and D-chiro-inositol use in PCOS treatment.

Study (first author, year)	MI and/or DCI dosage and duration	Main outcomes
Nestler, 1999 [38]	1200 mg of DCI/die versus placebo for six to eight weeks	In the group treater with DCI: (i) Area under the plasma insulin curve after the oral administration of glucose decreased (ii) Serum free testosterone concentration decreased (iii) Diastolic and systolic blood pressure decreased (iv) Plasma triglyceride concentrations decreased (v) Ovulatory rate increased

Laganà, 2015 [64]	1 gr of DCI/die plus 400 mcg of folic acid/die for 6 months	(i) Significant reduction of systolic blood pressure, Ferriman-Gallwey score, LH, LH/FSH ratio, total testosterone, free testosterone, Δ-4-androstenedione, prolactin, and HOMA index (ii) Significant increase of SHBG, glycaemia/IRI ratio, and menstrual cycle regularization

Pizzo, 2014 [65]	4 gr of myo-inositol/die plus 400 mcg of folic acid/die versus 1 gr of D-chiro-inositol/die plus 400 mcg of folic acid/die for six months	(i) MI compared to DCI decreased mostly systolic arterial pressure, LH/FSH ratio, total testosterone, D-4-androstenedione, prolactin, HOMA index, and, at the same time, SHBG considerably rises (ii) DCI compared to MI decreased more LH and free testosterone; at the same time, glycaemia/IRI ratio increased (iii) Both MI and DCI caused menstrual cycle regularization

Genazzani, 2008 [66]	MI 2 gr plus folic acid 200 mcg every day versus folic acid 200 mcg every day for 12 weeks	In the group treater with MI: (i) Plasma LH, prolactin, testosterone, insulin levels and LH/FSH resulted significantly reduced (ii) Insulin sensitivity expressed as glucose-to-insulin ratio and HOMA index resulted as significantly improved (iii) Menstrual cyclicity was restored in all amenorrheic and oligomenorrheic subjects

Gerli, 2003 [68]	100 mg, twice a day (=200 mg every day) of inositol (not specified if MI of DCI) versus placebo	(i) The ovulation frequency was significantly higher in the treated group compared with the placebo; the time in which the first ovulation occurred was significantly shorter (ii) The circulating concentration of E2 increased only in the inositol group during the first week of treatment (iii) Significant weight loss and leptin reduction were recorded in the inositol group (iv) Significant increase in circulating high density lipoprotein was observed only in the inositol treated group

Donà, 2012 [15]	MI 1200 mg/day versus placebo for 12 weeks	(i) MI treatment significantly improved metabolic and biochemical parameters (significant reductions were found in IR and serum values of androstenedione and testosterone) (ii) A significant association between band 3 tyrosine phosphorylation levels and insulin area under the curve was found at baseline but disappeared after MI treatment, while a correlation between band 3 tyrosine phosphorylation and testosterone levels was detected both before and after MI treatment

Nordio, 2012 [69]	550 mg MI + 13,8 mg DCI/day versus 2 gr MI/day for 3 months	(i) Plasma glucose and insulin concentrations showed a significant reduction in the MI + DCI group while no relevant changes were reported in the treatment with MI alone (ii) Compared to the MI group, the decrement of total testosterone and the increment of the serum SHBG were more relevant in MI + DCI group

Minozzi, 2013 [70]	550 mg MI + 13,8 mg DCI/day	(i) Improved LDL levels, HDL, triglycerides, and HOMA-IR.

MI: myo-inositol; DCI: D-chiro-inositol; LH: luteinizing hormone; FSH: follicle-stimulating hormone; SHBG: sex hormone binding globulin; E2: estradiol; HOMA: Homeostasis Model Assessment; IRI: glycaemia/immunoreactive insulin; IR: insulin-resistance; LDL: low density lipoprotein; HDL: high density lipoprotein.
